# Recommendations Emerging from Carbon Emissions Estimations of the Society for Neuroscience Annual Meeting

**DOI:** 10.1523/ENEURO.0476-22.2023

**Published:** 2023-10-12

**Authors:** Caroline Kay, Rob Kuper, Elizabeth A. Becker

**Affiliations:** 1 Department of Psychology, Saint Joseph’s University, Philadelphia, Pennsylvania 19131; 2 Department of Clinical Psychology, The Chicago School of Professional Psychology at Washington DC, Washington, DC 20005; 3 Department of Architecture and Environmental Design, Tyler School of Art and Architecture, Temple University, Ambler, Pennsylvania 19002; 4 Departments of Psychology and Neuroscience, Lawrence University, Appleton, Wisconsin 54911

**Keywords:** climate change, global warming, hybrid conference, multiple-site conference, virtual conference

## Abstract

The annual Society for Neuroscience (SfN) meeting yields significant, measurable impacts that conflict with the environmental commitment of the Society and the Intergovernmental Panel on Climate Change (IPCC) recommendations to address the climate emergency (IPCC, 2018). We used 12,761 presenters’ origins, two online carbon calculators, and benchmark values to estimate 2018 meeting-related travel, event venue operations, and hotel accommodation emissions. Presenters’ conference travel resulted in between 17,298 and 8690 tons of atmospheric carbon dioxide (t CO_2_), with or without radiative forcing index factors. Over 92% of authors traveled by air and were responsible for >99% of total travel-related emissions. Extrapolations based on 28,691 registrants yielded between 69,592.60 metric tons of carbon dioxide equivalents (t CO_2_e) and 38,010.85 t CO_2_ from travel. Comparatively, authors’ and registrants’ hotel accommodation emissions equaled 429 and 965 t CO_2_e, whereas operation of the San Diego Convention Center equaled ∼107 t CO_2_e. We relate SfN meeting-related emissions to potential September Arctic Sea ice loss, labor productivity loss in lower-income equatorial countries, and future temperature-related deaths. We estimate emissions reductions of between 23% and 78% by incentivizing between 10% and 50% of the most distant registrants to attend virtually or connecting between two and seven in-person hubs virtually. Completely virtual meetings may yield a reduction of >99% relative to centralized in-person meetings and increase participation of women, queer and transgender scientists, and scientists from low- and middle-income countries. We strongly recommend adopting alternative meeting modes such as four or more in-person global hubs connected virtually by 2030 and fully virtual by 2050.

## Significance Statement

“Rapid,” “far-reaching,” and “unprecedented” systems transitions that result in “deep [greenhouse gas] emissions reductions in all sectors” are needed to limit global warming to 1.5°C (IPCC, 2018, p 17). Our emissions estimations from the 2018 Society for Neuroscience conference venue, hotel accommodations, and travel indicate that most presenting authors traveled by air and emitted tens of thousands of tons of atmospheric carbon dioxide, as Aron et al. (2020) suggested. Among known impacts, SfN conference-related emissions likely yield measurable September Arctic Sea ice and labor productivity losses, and future temperature-related deaths. Our mathematical exploration of scenarios that reduce conference-related emissions that adhere to the Intergovernmental Panel on Climate Change reduction recommendations clearly and immediately impact the means of meeting and disseminating future work in neuroscience.

## Introduction

Between 2009 and 2019, Society for Neuroscience (SfN) annual meetings averaged 30,096 attendees from 77 countries (see Society for Neuroscience, “Attendance statistics: meeting attendance”; available at: https://www.sfn.org/meetings/attendance-statistics). Scientific conferences allow participants to exchange knowledge, techniques, and perspectives via presentation of current work and through formal and informal discussions, thus stimulating new relationships, ideas, and research (Parncutt et al., 2021). Yet, in-person meetings often exclude or marginalize individuals who identify as queer or transgender, require childcare or are lactating, face visa restrictions, and possess restricted travel budgets (Klöwer et al., 2020; Sarabipour et al., 2021; Fent et al., 2022; Gregor et al., 2023). Further, they rely on aircraft to convene, which accounted for 3.5% of net anthropogenic global warming in 2018 (Lee et al., 2021). Comparatively, hybrid, multihub, and fully virtual conferences reduce travel-related greenhouse gas (GHG) emissions (Parncutt et al., 2021) and have had higher attendance rates and greater representation by women, queer and trans individuals, and residents of low- and middle-income countries (Skiles et al., 2021; Yates et al., 2022).

In 2018, the Intergovernmental Panel on Climate Change (IPCC, 2018, p 14) recommended net anthropogenic carbon dioxide emission reductions of 45% by 2030, relative to 2010, and 100% by 2050 to limit the mean global surface temperature to 1.5°C above the preindustrial period (1850–1900) . The Sixth Assessment Report (IPCC, 2023, p 23) reiterates the need for “rapid, deep and in most cases immediate” emission reductions. Relative to 1850–1900, GHG emissions have increased the global surface temperature 1.1°C between 2011 and 2020 and driven sea-level rise, and food and water insecurity; human mortality from floods, droughts, and storms, and extreme heat events; and “substantial damages, and increasingly irreversible losses” in terrestrial, cryospheric, and aquatic ecosystems. Accordingly, systemic decarbonization is necessary.

Air travel accounts for a large portion of scientists’ annual emissions. A typical life sciences laboratory of 7-10 persons would “likely generate >20” tons of atmospheric carbon dioxide (t CO_2_) per year, or between 2 and 2.85 t CO_2_ per person (Nathans and Sterling, 2016). For comparison, one 13,000 km roundtrip flight may generate 1.3 t CO_2_. It has been reported (see “Towards a reduction of the carbon footprint at LOCEAN”; available at: https://www.researchgate.net/publication/348621541_Towards_a_reduction_of_the_carbon_footprint_at_LOCEAN) that laboratory function and numerical modeling each accounted for under one-quarter of 2018 emissions, whereas travel accounted for under half, of which 97% was from flights. In one PhD candidate case study, air travel accounted for 70% of total emissions. Forty-six percent of travel-related emissions was from conferences (Achten et al., 2013). Given that air transportation cannot derive comparable energy from fossil fuel alternatives (Smil, 2010, pp 228–230; Grote et al., 2014; Peeters et al., 2016; Lee et al., 2021) and with “no practical alternative in sight” to jet engines (Smil, 2010, p 224), scholarly norms that involve aircraft and institutional travel policies must change to decrease emissions in the scientific community.

Air travel accounts for most conference-related emissions (Tao et al., 2021). Booklets, programs, bags, and food all contributed <5% of conference-related emissions (Hischier and Hilty, 2002; Astudillo and Azari Jafari, 2018; Jäckle, 2019). Offering vegetarian or vegan catering menus could thus decrease emissions by only 1–2% (Astudillo and Azari Jafari, 2018; Tao et al., 2021). Hotel stays accounted for <10% (Neugebauer et al., 2020; Tao et al., 2021) to as little as 2% of the emissions impact (Astudillo and Azari Jafari, 2018). Finally, conference-related emissions from event venues and hotels equaled between 0.6% and 4.5% and 0.5–6.7%, respectively (Kuper, 2022).

Thus, we performed two estimations of travel-related carbon dioxide emissions. First, we computed 2018 SfN meeting event venue-, hotel-, and travel-related emissions. Second, we calculated and compared travel-related emissions from business-as-usual (BAU), hybrid, multihub, and virtual conferences to devise recommendations that address the organizational priority, diversity and inclusion, and environmental commitment of SfN, and allow SfN to meet the IPCC (2018) emissions reduction targets for 2030 and 2050.

## Materials and Methods

### Conference participants

We created a list of Neuroscience 2018 presenters from program PDFs on the SfN website, and extracted home institution, country, and city of residence for each of 12,825 presenters. For presentations with multiple authors, we extracted only emboldened presenting author names. We assumed that the percentages of authors by travel mode, and their mean travel-related emission weights, served as a proxy for the remaining 15,866 Neuroscience annual meeting registrants (*N* = 28,691). While our sample was substantial and likely encompassed a wide range of gender identities, we did not systematically collect or analyze data related to gender given that this information was not available in abstracts.

### 2018 Neuroscience conference

#### Travel-related emissions estimations using online calculators

We used directions from a web-mapping platform to determine travel times between presenting authors’ home city and San Diego, CA, the location of the 2018 SfN annual meeting, from which we classified presenters’ likely mode of travel as either car or air. Given that Southern California is not connected to an extensive railway system, compared with other US regions, we excluded this mode of transportation from our calculations. We deemed ≤2 h of travel time as drivable, which is approximately the time needed to travel by car between Los Angeles and San Diego. We assumed that on-road travel times >2 h resulted in air travel.

We used two web-based carbon calculators to estimate carbon dioxide emissions during presenting authors’ air travel to and from San Diego. While numerous online emissions calculators exist, we selected two with which we are familiar that would provide a range of values using slightly different calculation methodologies. The Carbon Footprint carbon calculator (available at: https://www.carbonfootprint.com/calculator.aspx), which uses a methodology and 2018 conversion factors defined by the United Kingdom, generated authors’ round-trip carbon dioxide emissions from the nearest airport to San Diego International Airport. The Carbon Fund business emissions calculators (available at: https://carbonfund.org/business-calculators/#gf_27) generated carbon dioxide emissions from presenters’ documented origins based on the number of miles, doubled for round-trip, between locations as reported by the web-mapping platform. Carbon Footprint calculations include a radiative forcing index (RFI) factor that accounts for an increased effect of global warming because of CO_2_ and non-CO_2_ emissions in the upper troposphere and lower stratosphere. We assumed that the Carbon Fund also used an RFI factor. Therefore, we divided aircraft emissions estimations by 1.891, the Carbon Footprint RFI factor, to yield estimates without radiative forcing. Our calculations are conservative estimates as they exclude the possibility of connecting flights, layovers, and any vehicular travel taken to and from the airports, hotels, and convention center. Thus, we assigned authors who originate from San Diego an emissions value of zero.

We also used Carbon Footprint and Carbon Fund to determine emission rates for car travel based on the most popular car to own in California in 2018, the USA 2018 Toyota Camry LE/SE SemiAuto-8 2WD Gasoline (see EVERQUOTE, “Most Common Cars in California vs. U.S.”; available at: https://www.everquote.com/california/buying-selling-autos/most-popular-cars/). The Carbon Footprint calculator returned emissions values for the Camry using mileage from authors’ origins to San Diego. The Carbon Fund calculator returned emissions values using gasoline fuel and the average highway miles per gallon (mpg) rate of 39 mpg for the 2018 Toyota Camry.

#### Cost of attendance

Following Skiles et al. (2021), we estimated registration, travel, lodging, and meal and incidental expenses (M & IEs) for the three rotating Society for Neuroscience meeting locations: San Diego, Washington DC, and Chicago. We included 2018 SfN meeting advance member registration fees ranging from $100 for undergraduate students to $400 for full members (see Society for Neuroscience, “Categories and fees”; available at: https://www.sfn.org/meetings/neuroscience-2018/registration/categories-and-fees).

We multiplied travel costs [in US dollars (USDs)] per kilometer for four flight zones in Dudás et al. (2016) by one-way great-circle distances between SfN authors’ origins and conference locations, which compared better to actual flight costs on a web-based travel metasearch engine than roundtrip cost estimates. We based travel costs by car on average costs per mile, converted to kilometers, over 15,000 annual miles as per AAA (American Automobile Association; “AAA’s your driving costs: how much does it cost to drive?”; https://exchange.aaa.com/automotive/aaas-your-driving-costs/). Because of varying rates of rail fare per kilometer in Europe (see Euronews.travel, “Rail fares across Europe: the countries with the most expensive train tickets”; available at: https://www.euronews.com/travel/2023/01/09/rail-fares-across-europe-the-countries-with-the-most-expensive-train-tickets) and lack of comparable information on rail fare rates per kilometer in the US, we assumed that train and car costs were equal. We derived per diem rates for lodging and M & IEs from the US General Services Administration for November 1 in fiscal year 2019, which were lower than actual 2018 SfN rates (see Society for Neuroscience, “Hotel list”; available at: https://www.sfn.org/meetings/neuroscience-2018/housing-and-travel/hotel-list). The sum of registration, travel, lodging, and M & IEs by transport mode represented attendance costs.

We examined attendance costs for parity for presenting authors who originated from developing countries. We derived 2018 gross domestic product (GDP) per capita in USDs for each World Bank category I, II, and III (low, lower-middle, and upper-middle income economies, respectively) country that authors represented from the International Monetary Fund World Economic Outlook Database (available at: https://www.imf.org/en/Publications/WEO/weo-database/2023/April/select-country-group). We calculated a mean GDP value for each economic category, like Skiles et al. (2021), which we compared with travel costs by travel mode.

#### Hotel accommodations

The 2020 Cornell Hotel Sustainability Benchmarking Study tool from the Hotel Carbon Measurement Initiative reported 2018 calendar year benchmark values where emissions per accommodation night per occupied room in an “urban location” of San Diego equaled 8.67 kg CO_2_e (Ricaurte and Jagarajan, 2020). We assumed that the presenting authors who originated from the San Diego area (*n *=* *390, 3.06%) did not require hotel stays. So, we multiplied emissions per night of stay by 4 nights (i.e., November 3–6), and the number of 2018 presenting authors (*n *=* *12,371) and total registrants (*n *=* *27,813), excluding local authors and registrants.

#### Event venue

Kuper (2022) reported 2019 electricity and natural gas use and total greenhouse gas emissions (5583.60 t CO_2_e) for the San Diego Convention Center derived from the City of San Diego, Department of Sustainability. We divided the total 2018 emissions by 365 d to yield a mean emissions rate per day, which we multiplied by 7, while assuming 1 d of setup and 1 d of disassembly before and after the 5 scheduled days of conference activity.

### Counterfactual travel-related emissions scenarios

The information that we gathered from presenting authors’ abstracts allowed us to explore potential travel-related emissions reductions of three different annual meeting scenarios. We assumed that the composition of authors, and by extrapolation, registrants, would mirror that of the 2018 conference; in reality, registrants’ origins and their travel-related emissions would differ from our results. The dataset is available for viewing and downloading here.

#### Multihub conference scenario

We compiled presenting authors’ origins by city, state, and country, and mapped and derived the latitude and longitude of each location in Google Earth. We then used spreadsheet software to compute the great-circle distance between authors’ origins and between one and seven conference locations (i.e., hubs) that would be connected virtually. As per Parncutt et al. (2021), to allow for 2–4 h of synchronous communication and shared content between hubs and for perceived equality among hubs, we selected two suites of three reference hubs that are spaced 7–9 h apart, as well as four additional hubs in each suite that are within 2 h of a reference hub. Furthermore, our hub selections accounted for concentrations of 2018 authors’ origins, connections to other airports (see OAG, “MEGAHUBS 2022: discover the most connected airports in the world”; available at: https://www.oag.com/megahub-airports-2022), and a desire to position one or more hubs in each suite in a developing country. We commanded software to identify the nearest hub and likely mode of travel for authors’ origins given calculated travel distances. We multiplied distances between authors’ origins and the nearest hub locations by the emission rates per travel mode in Jungbluth and Meili (2019). Aircraft emission rates in Jungbluth and Meili (2019) account for RFI factors and are derived from a review of five major approaches that were available and in practice in the 7 years before their study. In this scenario, we assumed that presenting authors would be encouraged and incentivized to prioritize overland rather than air travel and drive a car up to 4 h at an average rate of 60 miles/h, or ∼240 miles (400 km), at 180 grams (g) of atmospheric carbon dioxide equivalents (CO_2_e) per kilometer (km). Within 301–600 km, or 3 and 6 h away from cities where train travel is available, we assumed that presenting authors would travel by train at a rate of 50 g CO_2_e/km. We assumed that authors took short-haul flights for distances between 401 and 1500 km (see Klöwer et al.; available at: https://github.com/milankl/CarbonFootprintAGU) at rates of 230 and 340 grams g CO_2_e/km without and with RFI factors; medium-haul flights between 1501 and 4099 km (see EuroControl, “Data Snapshot: half of CO_2_ emissions come from just 6% of flights: the long haul ones; available at: https://www.eurocontrol.int/sites/default/files/2021-02/eurocontrol-data-snapshot-co2-by-distance.pdf) at 170 and 285 g CO_2_e/km; and long-haul flights for distances ≥4100 km at 118 and 230 g CO_2_e/km. We recognize that some travelers may tolerate longer train or car rides instead of short-haul flights, or prefer train travel over car travel for short distances, which would result in lower emissions than we have modeled. However, our estimations, again, assume direct flights and do not account for connections, which would inherently increase travel-related emissions (Astudillo and Azari Jafari, 2018).

In the multihub meeting scenario, for comparison, we used great-circle distances and emission rates from Jungbluth and Meili (2019) to calculate the travel-related emissions of the same Neuroscience 2018 presenting authors had they instead participated meetings in Chicago and Washington, DC, the other two centralized locations SfN uses as per their environmental commitment. In doing so, we assigned some authors whose nearest hub was Chicago to travel by train given the distance parameters outlined above.

In addition to estimating travel-related emissions based on Neuroscience 2018 presenting authors’ origins for multihub and virtual conference scenarios, we estimated travel-related emissions for the 28,691 registrants of Neuroscience 2018. We assumed that the percentages of presenting authors by travel mode and their mean rate of emission, relative to the nearest conference hub, could be applied to the larger sample of registrants and used to extrapolate their emissions estimations. Actual travel modes and emission rates would be different from our model.

#### Distance-dependent virtual participation (hybrid conferences)

To illustrate potential emissions reductions from a hybridized meeting model, we sorted our dataset from the online emissions calculators by travel-related emissions weight and removed 10%, 25%, and 50% of Neuroscience 2018 presenting authors who traveled the farthest and, consequently, emitted the most. For comparison, we also performed the same task using emission rates per kilometer from Jungbluth and Meili (2019) and great-circle distances between the latitudes and longitudes of authors’ origins and San Diego, Washington DC, and Chicago. The sum of each scenario represents conferences where these presenting authors would be incentivized to attend virtually only via reduced registration rates.

#### Virtual conference

We used formulae Jäckle (2021) presented to estimate emissions from the operation of attendees’ devices and transfer of audiovisual information related to virtual meetings. We inserted an electricity generation rate of 0.85 pounds/kWh (see U.S. Energy Information Agency, “Frequently asked questions: how much carbon dioxide is produced per kilowatthour of U.S. Electricity generation?”; available at: https://www.eia.gov/tools/faqs/faq.php?id=74&t=11) and 60 active conference hours between November 2 and 7, which include posters, lectures, roundtables, workshops, and symposia, and exclude evening socials and receptions. Data usage per hour of video teleconferencing, electricity usage per watt, and attendance percentages varied, as per Jäckle (2021). We performed the calculations twice, once with unique presenting authors and once with total registrants (i.e., *N *=* *12,761 and 28,691).

## Results

### 2018 Neuroscience conference

**Figure 1.**
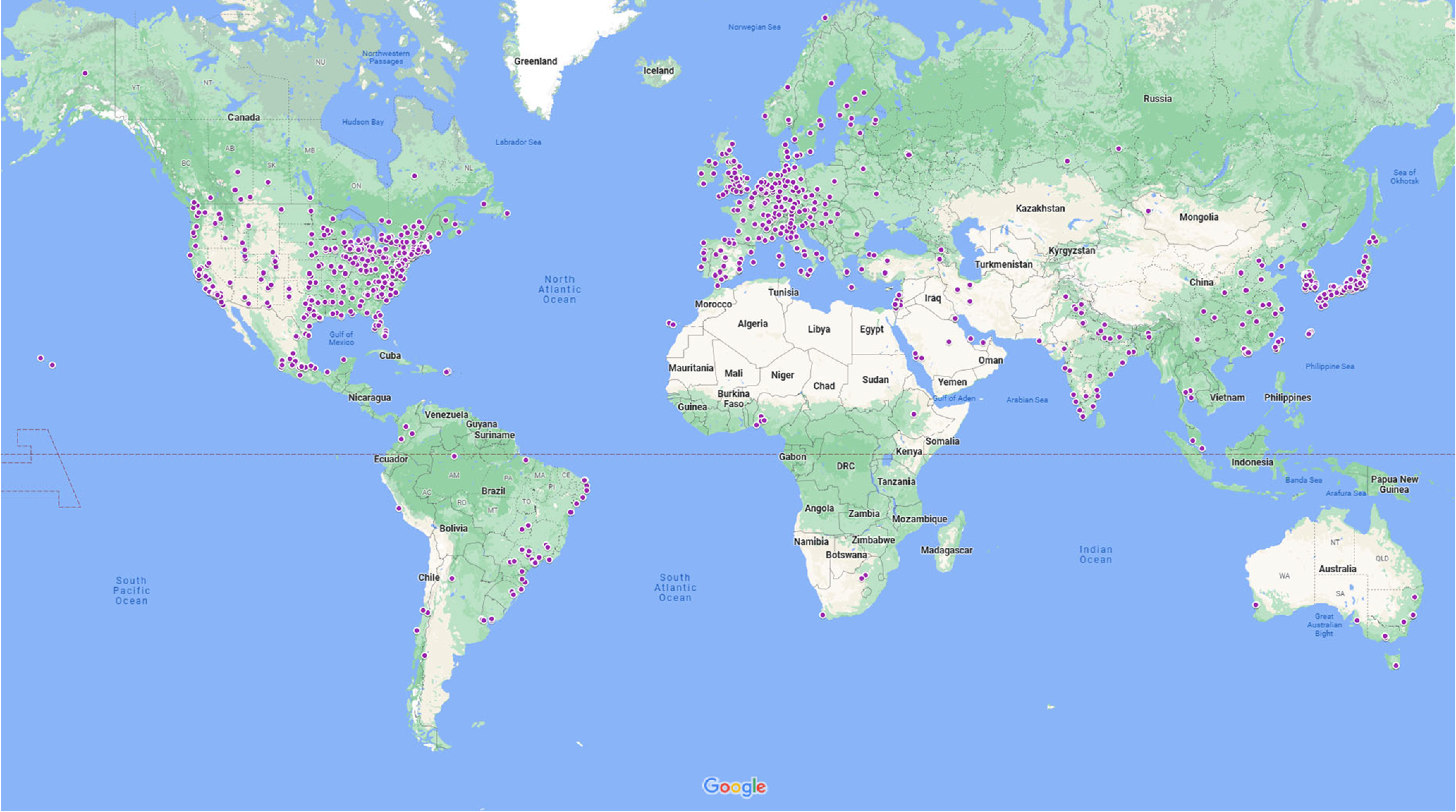
Neuroscience 2018 presenting authors’ global origins.

The 2018 Society for Neuroscience conference included 22,726 scientific and 5965 nonscientific registrants. Of 12,825 presenting authors we cataloged, 63 presented more than once and one author’s origin could not be identified; our dataset included 12,761 unique presenting authors (Table 1). Presenting authors originated from 69 countries and 1208 different towns (Fig. 1). Among the types of organizations, hospitals accounted for >8% of institutions; research institutions accounted for almost 11%; companies and organizations accounted for >13%; and universities, colleges, graduate, and medical schools accounted for the largest portion of almost 66%. Of the 1616 organizations represented, 54% (*n *=* *877) were public entities, 31% (*n *=* *503) were private institutions, 7% (*n *=* *115) were government funded, and 6% (*n *=* *99) were nonprofit organizations.

**Table 1:** 2018 SfN Presenting authors’ travel-related emissions, in metric tons of carbon dioxide (t CO_2_), by organization type

Organization categories/type	Authors	Unique organizations	Carbon Footprint with RFI	Carbon Footprint	Carbon Fund with RFI	Carbon Fund
Hospital	465	137	600.04	317.81	586.07	309.69
Government-funded	51	27	42.31	22.43	41.69	22.10
Nonprofit	104	24	74.70	39.87	78.04	41.44
Private	184	32	236.33	125.13	236.88	124.89
Public	126	54	246.70	130.38	229.46	121.26
Research institute	1227	173	2078.28	1099.11	1936.21	1024.06
Government-funded	588	74	1097.36	579.99	1024.78	542.09
Nonprofit	441	56	534.25	282.93	504.75	266.92
Private	61	32	123.24	65.27	114.59	60.59
Public	137	30	323.43	170.92	292.09	154.46
University/college/school	10,561	1063	13,849.26	7334.30	13,185.63	6976.52
Government-funded	18	3	24.68	13.00	24.56	12.97
Private	3269	331	3853.63	2042.57	3794.55	2006.21
Public	7274	729	9970.95	5278.73	9366.52	4957.34
Organization/company	439	221	614.51	325.56	574.89	304.02
Government-funded	55	11	101.64	53.78	93.17	49.26
Nonprofit	29	19	39.26	20.82	38.70	20.48
Private	199	127	281.77	149.23	261.66	138.46
Public	156	64	191.84	101.73	181.36	95.82
Miscellaneous	62	14	142.54	75.38	130.17	68.75
Unlisted	5	5	10.97	5.80	9.86	5.21
N/A	3	3	2.32	1.23	2.44	1.29
Totals	12,762	1616	17,297.92	9159.19	16,425.27	8689.54

N/A, Not applicable. Radiative forcing index (RFI) factors account for an increased effect of global warming because of CO_2_ and non-CO_2_ emissions in the upper troposphere and lower stratosphere. The miscellaneous category includes one business school, one dual program spanning public/private universities, an independent charity, independent laboratory, international association, two learned societies, one nonprofit university foundation, a private foundation, private high school, private international school, public charity, public high school, and one scientific cooperation foundation. Unlisted organizations included a hospital, a company, and three research institutes.

Neuroscience 2018 presenting authors mostly represented the global north (Table 2). Over 68% of presenters (*n *=* *8782) originated from North America and close to 16% originated from Europe, Western Asia, and the Middle East (*n *=* *2009). Just >13% of presenters originated from Asia, New Zealand, and Australia (*n *=* *1711). About 2% of presenting authors originated from South America and Africa. Overall, the vast majority of presenters originated from the US; consequently, the US is responsible for most of the travel-related emissions. Within the US, including nearby Canadian provinces, about one-quarter of presenting authors originated from the Mid-Atlantic region (*n *=* *2159), 18% hailed from California, and 16% originated from New England (Extended Data Table 2-1).

**Table 2:** Neuroscience 2018 presenting authors’ travel-related emissions, in t CO_2_, by continent and country or region (*N *=* *12,761)

Continent	Country/region	Countries	Carbon Footprint with RFI	Carbon Footprint	Carbon Fund with RFI	Carbon Fund	Presenters
North America	United States	1	5528.18	2935.03	5764.71	3051.80	7748
Europe	Western Europe	16	4574.35	2419.60	4046.53	2140.00	1764
Asia	East Asia	7	3828.07	2024.39	3417.38	1807.59	1410
North America	Canada	1	619.03	327.86	659.39	347.75	686
Asia	South Asia	2	507.83	268.61	460.32	243.38	132
South America	Northern South America	3	392.89	207.63	365.58	193.40	157
Asia	Middle East	11	374.74	197.90	347.30	183.78	111
Australia	Australia	1	363.37	192.13	325.61	172.22	107
Europe	Eastern Europe	11	309.79	163.69	278.32	147.16	113
South America	Southern South America	3	228.76	121.03	206.09	109.06	89
Latin America	Mexico	1	207.05	108.80	221.97	117.46	328
Asia	Southeast Asia	3	136.85	72.38	124.13	65.72	35
New Zealand	Polynesia	1	60.78	32.15	56.53	29.91	20
Asia	Siberia	1	54.58	28.88	43.61	26.17	20
Africa	Southern Africa	1	35.88	18.99	31.91	16.88	8
North America	Caribbean	2	29.08	15.43	26.31	13.95	20
Asia	Central Asia	1	26.06	13.79	24.24	12.83	7
Africa	West Africa	1	13.76	7.27	12.56	6.64	4
Africa	East Africa	1	4.10	2.17	4.21	2.23	1
Africa	North Africa	1	2.68	1.42	2.39	1.26	1

RFI factors account for an increased effect of global warming because of CO_2_ and non-CO_2_ emissions in the upper troposphere and lower stratosphere. Carbon footprint uses an radiative forcing index (RFI) factor of 1.891, which we assumed carbon fund to use as well. Extended Data Table 2-1 presents Neuroscience 2018 annual meeting US and Canadian presenting authors by region (*n *=* *8278).

As of April 2023, SfN claimed “>36,000 members in >95 countries” (see Society for Neuroscience, “Directories”; available at: https://my.sfn.org/Directories/Individual-Members). The individual member directory contains 797 SfN members residing in two category I (2 members), 11 category II (99 members), and 20 category III (696 members) countries. Thus, >35,203 SfN members (97.79%) are registered in high-income countries. In our 2018 dataset, 957 total authors represented 1 category I (1 author), 4 category II (146 authors), and 16 category III (810 authors) developing countries. Authors mostly represented Mexico (*n *=* *328), China (*n *=* *269), Brazil (*n* =143), and India (*n *=* *131). Thus, 11,804 (92.50%) of the presenting authors originated from high-income countries.

#### Costs of attendance

**Figure 2.**
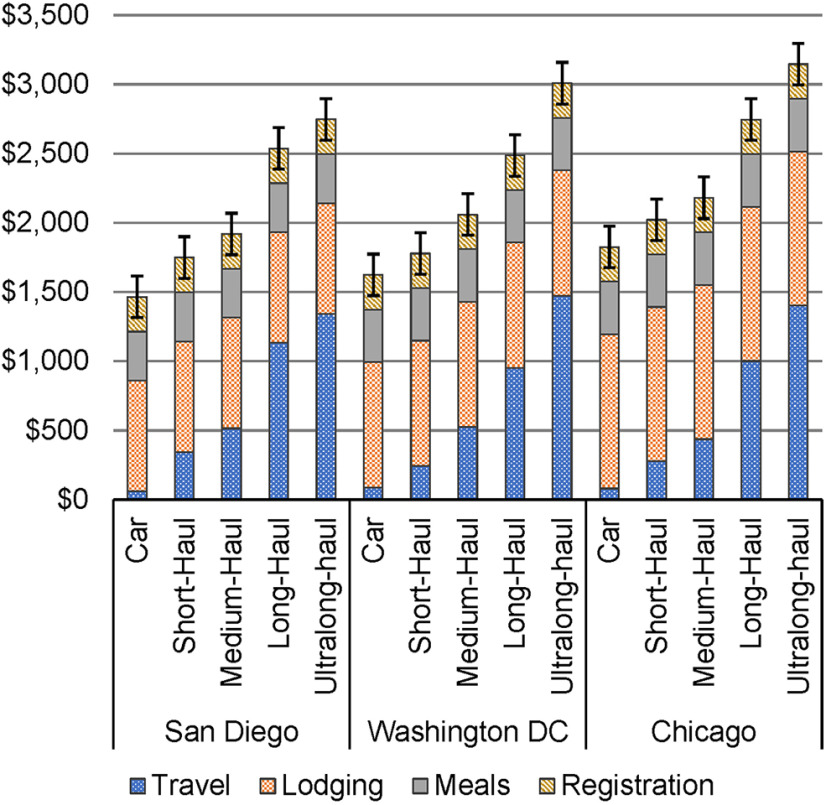
Costs of attending current, centralized annual Neuroscience meetings in USDs. Error bars represent registration costs from $100 to $400 that are associated with membership status.

We found SfN attendance costs to be greatest for Chicago ($1676 to $3296) and least expensive for San Diego ($1314 to $2898; Fig. 2). Regardless of BAU conference location, the further one travels, the greater the financial cost. Lodging accounts for the greatest proportion of attendance costs for overland and short-haul and medium-haul air travelers, whereas travel costs account for the greatest proportion of long-haul and ultra-long-haul air travelers. The cost of attending an SfN conference was between 1.56 and almost four times the mean GDP per capita for an author originating from a category I country (mean = $839.86); between 64% and 1.59 times the GDP per capita in a category II ($2067.65) country; and between 13.84% and 34.71% of the GDP per capita in a category III ($9496.89) country. Comparatively, attendance costs for a US resident equal between 2.09% and 5.25% of the 2018 GDP per capita ($62,787.78).

10.1523/ENEURO.0476-22.2023.tab2-1 Table 2-1 Neuroscience 2018 U.S. and Canadian presenting authors by region (*n *=* *8278). Download Table 2-1, DOC file.

#### Travel-related emissions

With the use of online carbon dioxide emissions calculators (i.e., Carbon Footprint and Carbon Fund), we estimated that travel-related carbon dioxide emissions to San Diego equaled between 17,298 and 16,425 metric tons of atmospheric carbon dioxide equivalents (t CO_2_e), which account for RFI factors, and between 9159 and 8690 t CO_2_ without radiative forcing. Over 92% of presenting authors (*n *=* *11,797) traveled by aircraft and were responsible for between 99.89% and 99.79% of travel-related emissions, whereas the remaining ∼7.5% of presenters (*n *=* *964) who traveled by car were responsible for <0.5% of emissions, or >18 t CO_2_. Put another way, there were >12 times more air travelers than car travelers, but the former emitted between 463 and 476 times more travel-related carbon dioxide than the latter, and as much as 900 times more when accounting for CO_2_ and non-CO_2_ emissions. On average, a car traveler emitted 0.01–0.02 t CO_2_ to travel to San Diego; an air traveler emitted 37 or 73 times more (i.e., 0.74 or 1.46 t CO_2_e), depending on whether additional effects of non-CO_2_ aircraft emissions in the upper atmosphere are included.

#### Hotel accommodations

Neuroscience 2018 presenting authors and registrants who traveled from beyond the San Diego region may have been responsible for emitting 429.02 and 964.55 t CO_2_e for 4 nights of hotel stays. Registrants aside, emissions from presenting authors’ hotel stays are 23 times that of travel-related emissions from cars, but between 2.5% and ∼5% of travel-related emissions from aircraft.

#### Event venue

SfN may be responsible for the emissions of 107.08 t CO_2_e by convening in the San Diego Convention Center for 7 d in 2018. Each of 28,691 registrants could be responsible for 0.00373 t CO_2_e, or 3.73 kg CO_2_e for use of the event venue across 7 d, which is 43% of a hotel stay for a single night.

### Counterfactual travel-related emissions scenarios

#### Multihub conference

**Figure 3.**
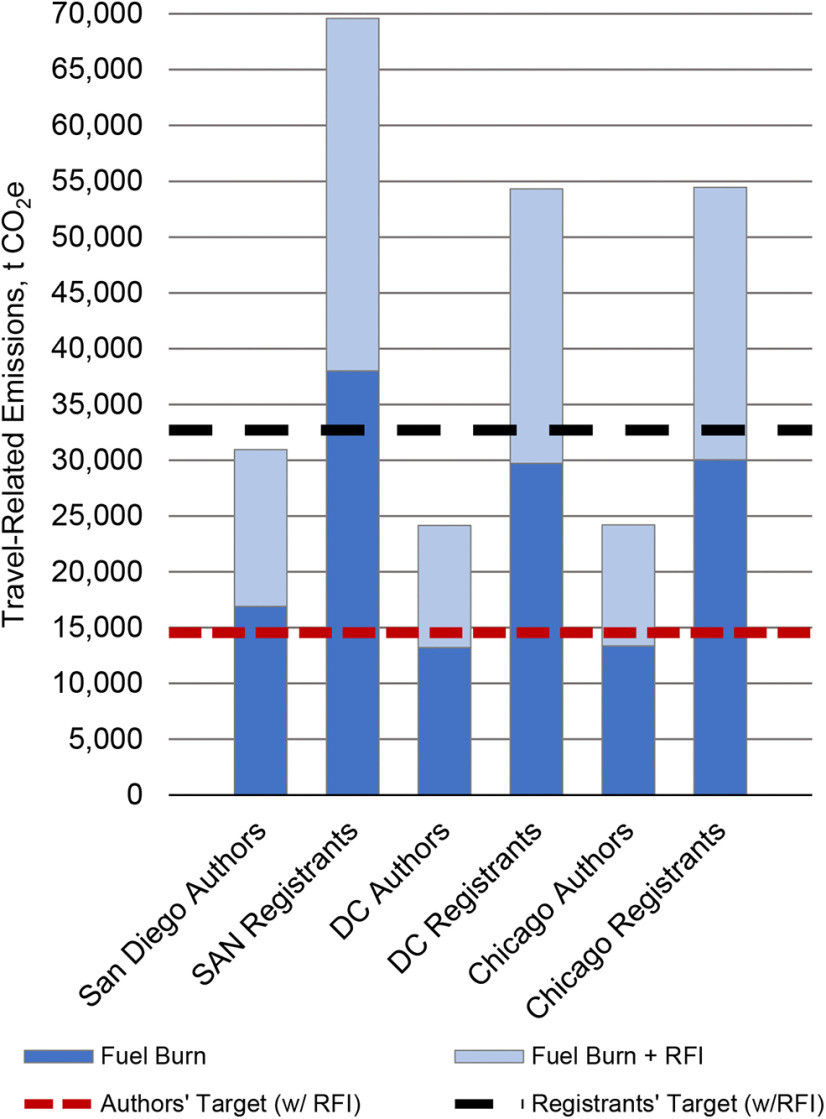
Travel-related emissions estimations, presented in metric tons of atmospheric carbon dioxide equivalents (t CO_2_e), derived from great-circle distances between Neuroscience 2018 presenting authors’ (*N *=* *12,761) latitudes and longitudes and current, centralized meeting locations using emissions rates from Jungbluth and Meili (2019). Also included are extrapolations for 28,691 registrants derived from mean emissions rates and percentages of Neuroscience 2018 presenting authors by travel mode. Radiative forcing index (RFI) factors account for an increased effect of global warming because of CO_2_ and non-CO_2_ emissions in the upper troposphere and lower stratosphere. The 45% emissions reduction targets are relative to the mean travel-related emissions estimation across the three business-as-usual in-person meeting locations, San Diego, Washington, DC, and Chicago. Including emissions from connecting flights could increase emissions more than shown. Extended Data Figure 3-1 presents the travel-related emissions presented here by travel mode.

Travel-related emissions to the San Diego SfN conference using presenting authors’ departure latitude and longitude and emission rates from Jungbluth and Meili (2019) were higher than those for travel from to Washington, DC, and Chicago, the other two BAU conference locations (Fig. 3). In each case, long-haul flight travel (≥4100 km) accounted for between 63% and 68% of emissions, whereas overland travel accounted for <0.15% on average (Extended Data Fig. 3-1).

10.1523/ENEURO.0476-22.2023.f3-1 Figure 3-1 Travel-related emissions estimations, presented in t CO_2_e, derived from great-circle distances between Neuroscience 2018 presenting authors’ (*N *=* *12,761) latitudes and longitudes and current, centralized meeting locations using emission rates from Jungbluth and Meili (2019). Also included are extrapolations for 28,691 registrants derived from mean emission rates and percentages of Neuroscience 2018 presenting authors by travel mode. Emission reduction targets of 45% are relative to mean travel-related emissions estimation across the San Diego, Washington, DC, and Chicago conference locations. Estimates for air travel include RFI factors that account for non-CO_2_ emissions, but do not include connecting flights, which could increase emissions more than shown. Authors’ emissions from cars for the San Diego, Washington, DC, and Chicago conference venues equaled 38.71, 45.17, and 20 t CO_2_, respectively; registrants’ emissions equaled 87.03, 101.56, and 44.96 t CO_2_, respectively. Authors’ emissions associated with train travel to Washington, DC and Chicago equaled 73.01 and 32.06 t CO_2_e; registrants’ emissions equaled 165.50 and 73.44 t CO_2_e. Download Figure 3-1, TIF file.

**Figure 4.**
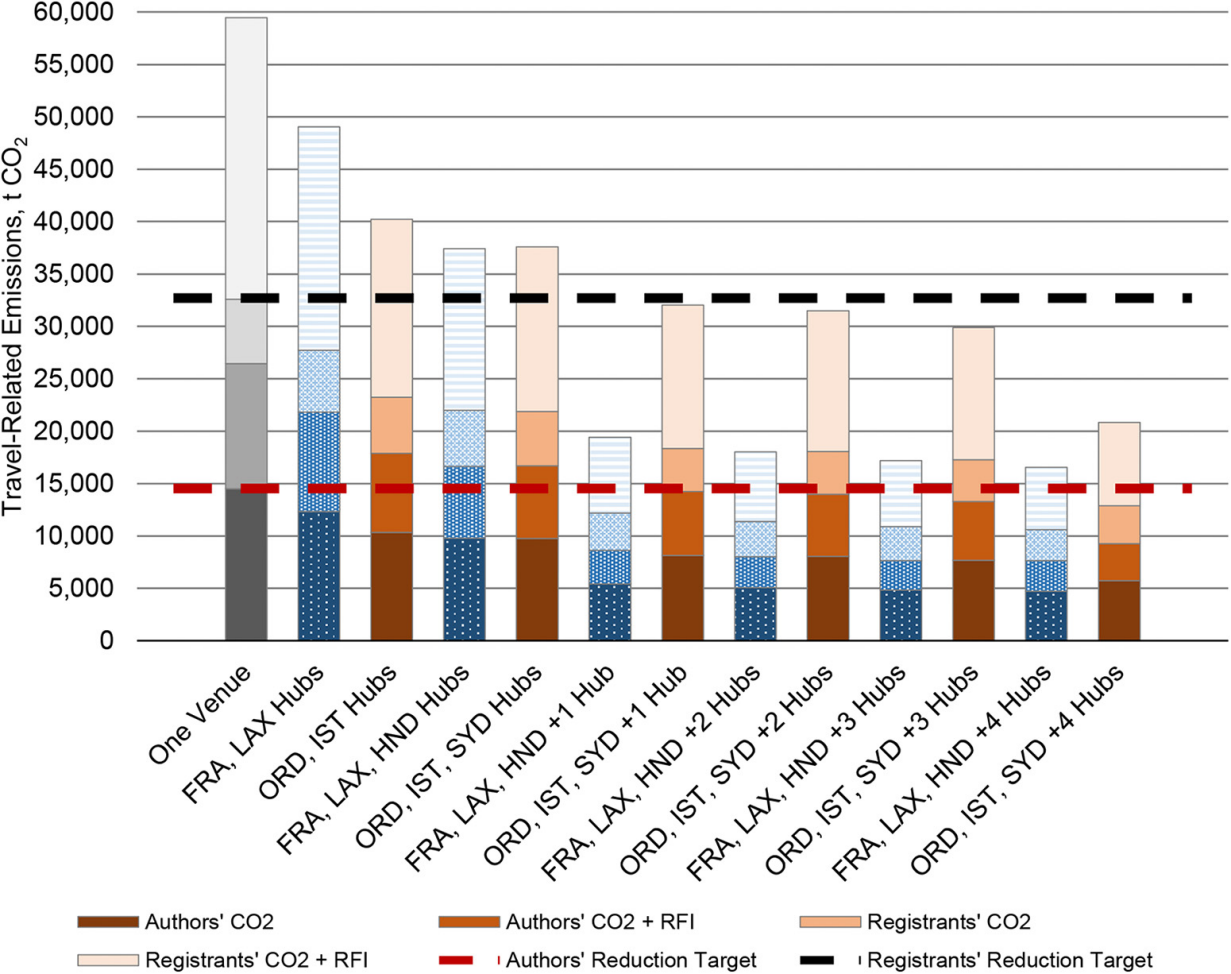
Travel-related emissions estimations, presented in t CO_2_, of multihub suites relative to the mean emissions estimation from San Diego, Washington, DC, and Chicago meeting locations. All values are computed using great-circle distances between Neuroscience 2018 presenting authors’ origins and meeting locations and emission rates from Jungbluth and Meili (2019). Radiative forcing index (RFI) factors account for an increased effect of global warming because of CO_2_ and non-CO_2_ emissions in the upper troposphere and lower stratosphere. One multihub suite contains the three reference hubs of Frankfurt (FRA), Los Angeles (LAX), and Tokyo (HND). Thereafter, we added, in order, Chicago (ORD), Dallas/Ft. Worth (DFW), Mexico City, and Jakarta. The second suite contained ORD, Istanbul (IST), and Sydney (SYD), to which we added Philadelphia, Riyadh, São Paulo, and HND. We did not account for connecting flights, which could increase emissions more than shown. Extended Data Figures 4-1 and 4-2 present counterfactual multihub meeting travel-related emissions by travel mode, hub scenario, and location that we derived from Neuroscience 2018 presenting authors (*N *=* *12,761) for the suite of hubs that include FRA, LAX, and HND.

Though both multihub suites yielded travel-related emissions reductions of at least 45%, relative to our BAU mean, by deploying four hubs, the suite that included Frankfurt, Germany; Los Angeles; and Tokyo as reference hubs yielded greater emissions reductions than that of Chicago, Istanbul, and Sydney (Fig. 4). Hence, we report on the former suite only, which decreased authors’ travel-related emissions across two and seven hubs by ∼17.5–72% relative to the mean of the three BAU locations (mean* *=* *26,442.50 t CO_2_e). The largest emissions reductions occurred between two and three hubs, and three and four hubs (37% and 67%, respectively, relative to BAU), whereupon presenters’ reliance on long-haul flights dropped from 44% to 23% (Extended Data Figs. 4-1, 4-2). Thereafter, the law of diminishing returns becomes evident; emissions reductions equal between 2.33% and 1%. Moreover, even with seven hubs, ∼58% of presenting authors would still rely on short-haul flights, while 21% and 18% would rely on medium-haul and long-haul flights. Presenters’ numerous widespread origins prevent a shift toward predominately overland travel.

10.1523/ENEURO.0476-22.2023.f4-1 Figure 4-1 Counterfactual multihub meeting travel-related emissions, presented in metric tons of carbon dioxide equivalents (t CO_2_e), by travel mode, hub scenario and location derived from Neuroscience 2018 presenting authors (*N *=* *12,761). Estimates include RFI factors that account for non-CO_2_ aircraft emissions. We did not account for connecting flights, which could increase emissions more than shown. Download Figure 4-1, TIF file.

10.1523/ENEURO.0476-22.2023.f4-2 Figure 4-2 Summary of counterfactual multihub meeting travel-related emissions, presented in metric tons of carbon dioxide equivalents (t CO_2_e), by hub scenario and travel mode derived from Neuroscience 2018 presenting authors (*N *=* *12,761). Estimates include RFI factors that account for non-CO_2_ aircraft emissions. Three reference hubs include Frankfurt, Los Angeles, and Tokyo. Thereafter, we added, in order, Chicago, Dallas/Fort Worth, Mexico City, and Jakarta. We did not account for connecting flights, which could increase emissions more than shown. Download Figure 4-2, TIF file.

Travel costs decreased with travel distance, the greatest of which occurred because of travel mode shifts. Short-haul travel costs decreased, on average, $2 between one and seven hubs. Medium-, long-, and ultra-long-haul travel costs decreased $13, $59, and $234, respectively. Shifting from one travel mode to another between one and seven hubs dropped travel costs, on average, $162 (medium- to short-haul air travel) to $409 (long- to medium-haul air travel).

**Figure 5.**
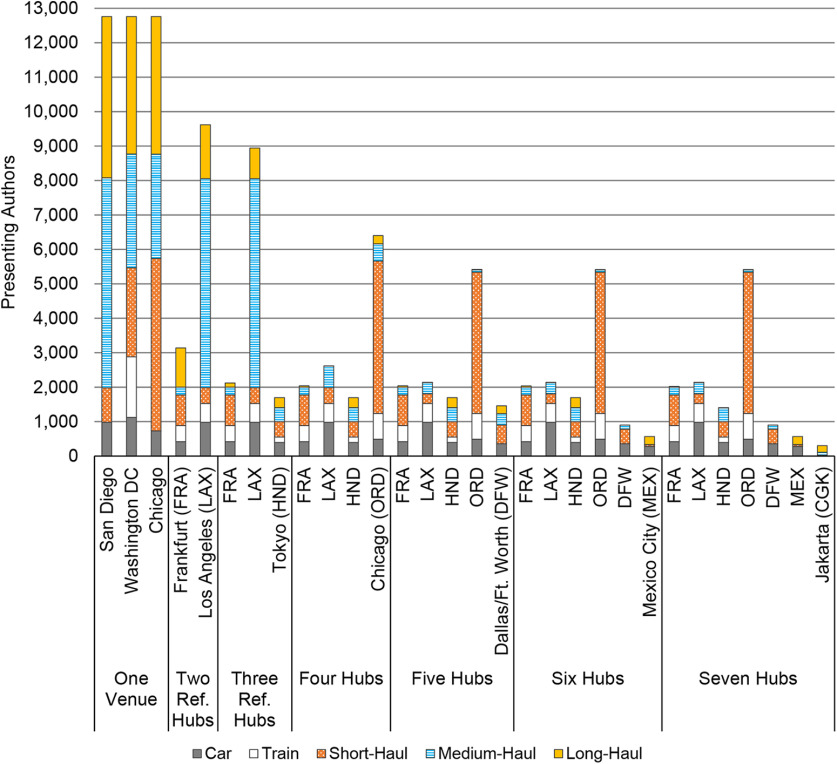
Neuroscience 2018 presenting author (*N *=* *12,761) distribution by travel mode, hub scenario, and location for counterfactual multihub meetings. Reference hubs are spaced 7–9 h apart in coordinated universal time. Four additional hubs are spaced 1 or 2 h apart from a reference hub. FRA, LAX, and HND coordinated universal times are +2, −7, and +9, respectively. LAX serves as the reference hub for ORD, DFW, and Mexico City International Airport (MEX). CGK is the reference hub for HND. Extended Data Figure 5-1 presents Neuroscience 2018 presenting authors (*n *=* *957) who originated from a developing country by World Bank income category in Fiscal Year 2019, hub scenario, and location.

The number of presenting authors across multihub models is consistently unequal (Fig. 5). With two hubs, Los Angeles International Airport (LAX) served as the closest hub for 75% of presenting authors. Although 20% of authors traveled to LAX in the four-hub model, Chicago O’Hare International Airport (ORD) became the nearest hub for >50% of authors. Chicago remained the largest hub when we deployed five, six, and seven hubs (42%); the three reference hubs averaged 15% of authors, and the two hubs in developing countries averaged only 4.63% of authors. Finally, with five or more hubs, authors originating from developing countries were almost absent in Chicago and Dallas/Fort Worth [DFW; and Frankfurt (FRA) with six or more hubs; Extended Data Fig. 5-1].

10.1523/ENEURO.0476-22.2023.f5-1 Figure 5-1 Attendance of Neuroscience 2018 presenting authors (*n *=* *957) who originated from a developing country by World Bank income category in FY 2019, hub scenario, and location. Download Figure 5-1, TIF file.

#### Distance-dependent virtual participation (hybrid conferences)

**Figure 6.**
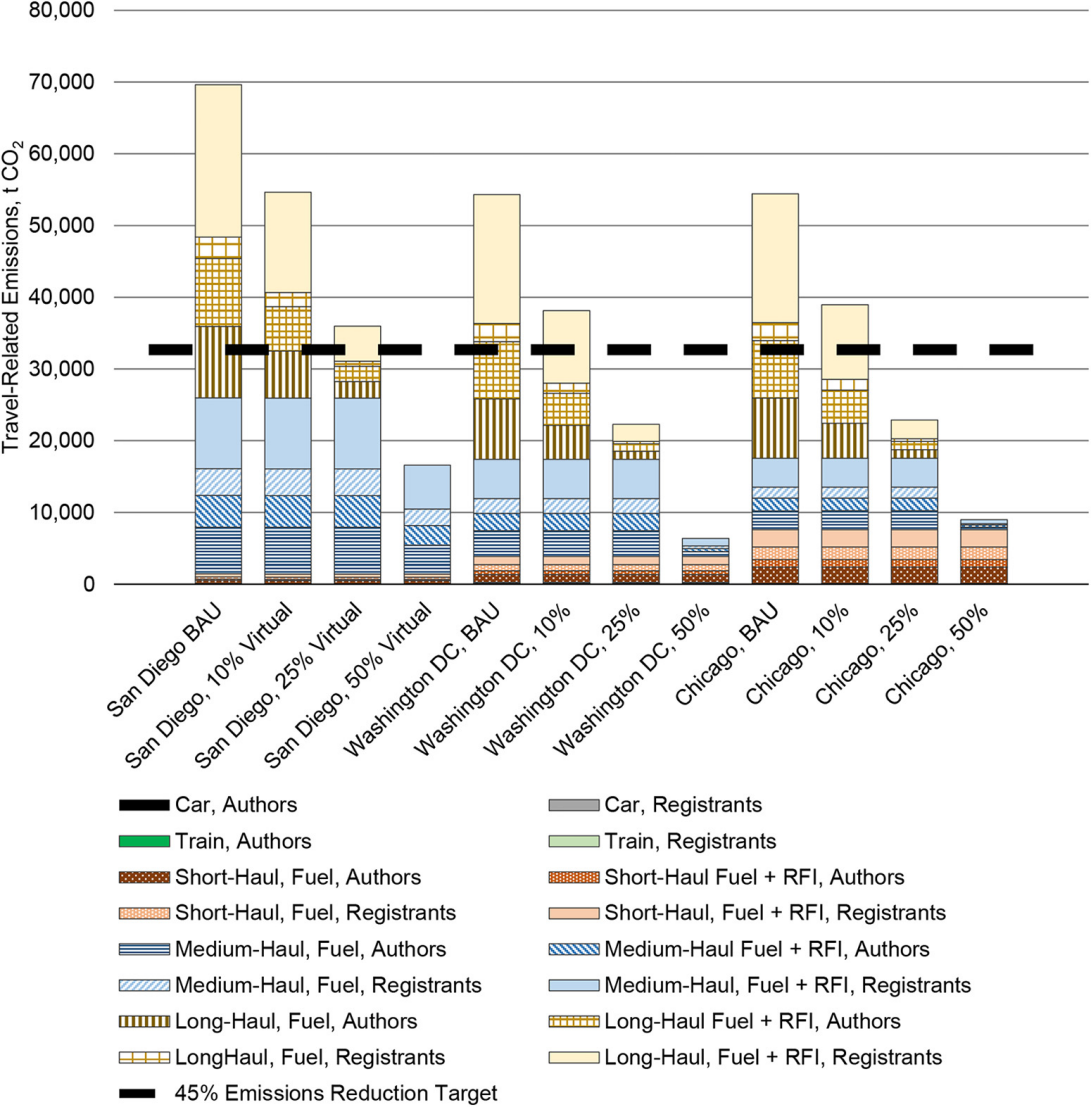
Travel-related emissions, presented in t CO_2_, for business-as-usual (BAU) and hybridized Neuroscience meetings when 10%, 25%, and 50% of the most distant attendees participate virtually. All values derived from emission rates per kilometer in Jungbluth and Meili (2019) and relative to BAU in-person meetings. The emissions reduction target accounts for radiative forcing index (RFI) factors and is relative to the mean travel-related emissions across BAU sites. We did not account for connecting flights, which could increase emissions more than shown.

Using values from online calculators, 10%, 25%, and 50% virtual participation of Neuroscience 2018 presenting authors yielded emission reductions of 23%, between 49% and 52% (with and without RFI factors), and between 76% and 78%, respectively. We found similar reductions for San Diego in the counterfactual scenarios derived from rates in Jungbluth and Meili (2019). Emissions reductions for Washington, DC, and Chicago equaled ∼28%, 58%, and between 83% and 88% when 10%, 25%, and 50% of the most distant authors attended virtually; in the latter, long-haul flights were eliminated and emissions from medium-haul flights were reduced >80% (Fig. 6).

#### Virtual meetings

Emissions estimates resulting from the transfer of audio and visual data and device consumption only for presenting authors yielded travel-related emissions reductions of between 99.54% and 99.98%, depending on the BAU conference location used for comparison. Reductions of between 99.95% and 98.96% may be possible when accounting for 28,691 registrants.

## Discussion

### Neuroscience 2018 conference

Our total carbon emissions estimates from the 2018 SfN meeting equal “tens of thousands of tons of CO_2_ for flights alone,” as Aron et al. (2020) estimated, surpassing the figures reported by Nathans and Sterling (2016) by 2.7 to >3 times, possibly because we accounted for RFI factors. Air travel-related emissions that account for RFI factors and were derived from online carbon calculators account for over 97% of our estimates. Emissions from hotel accommodations, use of the San Diego Convention Center, and car travel accounted for 2.41%, 0.27%, and 0.07% of our estimates, respectively, which are lower than estimates reported by Neugebauer et al. (2020) and Kuper (2022), likely because of our larger sample size and methodological differences. Among known impacts, SfN conference-related emissions translate to 3 m^2^ (∼4 × 8 feet in area) of September Arctic Sea ice loss for every t CO_2_ emitted, which contributes to further sea ice loss by absorbing solar radiation and increasing ocean temperatures (Stroeve and Notz, 2018); labor productivity losses equal to $0.59 (2011 USDs; $0.79 per t CO_2_ in 2023 USDs) in agricultural, construction, manufacturing, mining, and quarrying sectors across lower-income, equatorial regions because of warmer, wetter air and laborers’ need to take more frequent breaks to avoid heat exhaustion (Chavaillaz et al., 2019). For every 4434 t CO_2_ emitted, without accounting for infectious disease, civil and interstate war, food supply, and flooding related to climate change, one excess temperature-related death somewhere in the world between 2020 and 2100 will occur (Bressler, 2021).

We show that the largest sector of Neuroscience 2018 presenters represented universities, colleges, or schools (*n *=* *10,561), which must contribute to the transition toward alternative conference meeting modes. Institutions and grant funders should measure, report, and set aggressive, absolute travel-related emissions reduction targets that meet IPCC recommendations [e.g., 50% by 2025, and 75% by 2030, as Hoolohan et al. (2021) reported for one university], prioritize funding for “virtual conferencing hardware and low-carbon forms of travel” (Bousema et al., 2022), and require justification for air travel based on the value and impact of proposed activities relative to career stage (see “Towards a culture of low-carbon research for the 21st century. Tyndall Working Paper 161”; available at: https://tyndall.ac.uk/wp-content/uploads/2021/09/TWP-161.pdf). Some universities have implemented carbon prices on air travel that range from $0.01 per mile to $40 USDs per trip, which can be used to fund sustainable university projects but fail to alter existing travel policies and behaviors (Barron et al., 2020). Instead, carbon prices must at least equal the social cost of carbon ($51 per t CO_2_ in US), though Rennert et al. (2022) found that $185 per t CO_2_ better reflects some risks of climate damage. For comparison, using direct air capture to remove 1 t CO_2_ costs $750 to $1000 (see “The quest to trap carbon in stone—and beat climate change”; available at: https://www.wired.com/story/the-quest-to-trap-carbon-in-stone-and-beat-climate-change/), or ∼2 to 2.5 times the airfare of a roundtrip flight, without the RFI factor, between Charleston, SC, and San Diego.

Our estimates portray only a portion of Neuroscience 2018 meeting-related emissions. We did not estimate emissions related to ground transportation; printing of programs and other materials; meals and drinks; water consumption; information and communication technology; and wastewater and waste treatment, which have measurable environmental impacts (Neugebauer et al., 2020; Tao et al., 2021). The operation of equipment (e.g., floor sweepers, forklifts, carts), and the assembly, disassembly, production, and transportation of exhibit signage, podiums, and stages could also be estimated (Toniolo et al., 2017). Thus, more comprehensive meeting-related emissions estimates would be even greater than those we present, though travel would still likely account for most (Neugebauer et al., 2020; Tao et al., 2021).

### Future neuroscience conferences

SfN must alter the annual meeting format and, like other organizations, “cannot simply purchase [their] way to climate action” (Gifford, 2022). Buying “cheap credits [i.e., offsets] that often lack integrity instead of immediately cutting their own emissions,” may constitute greenwashing (see “Integrity matters: net zero commitments by businesses, financial institutions, cities and regions”; available at: https://www.un.org/sites/un2.un.org/files/high-level_expert_group_n7b.pdf?%20Financial%20Institutions,%20Cities%20and%20Regions). Furthermore, carbon offsets (e.g., planting trees) do not encourage behavioral changes that contribute to low-carbon lifestyles, and may erode support for carbon taxes, which will more effectively reduce emissions (Werfel, 2017; Hagmann et al., 2019). Moreover, offsets may displace undesirable practices to less regulated locations, encourage rising emissions for greater potential future reductions and offsets, fall victim to conflicts of interest, may not meet the essential criteria of additionality (i.e., projects may double-count emissions reductions), and may not permanently prevent or remove atmospheric emissions (McAfee, 2022).

**Figure 7.**
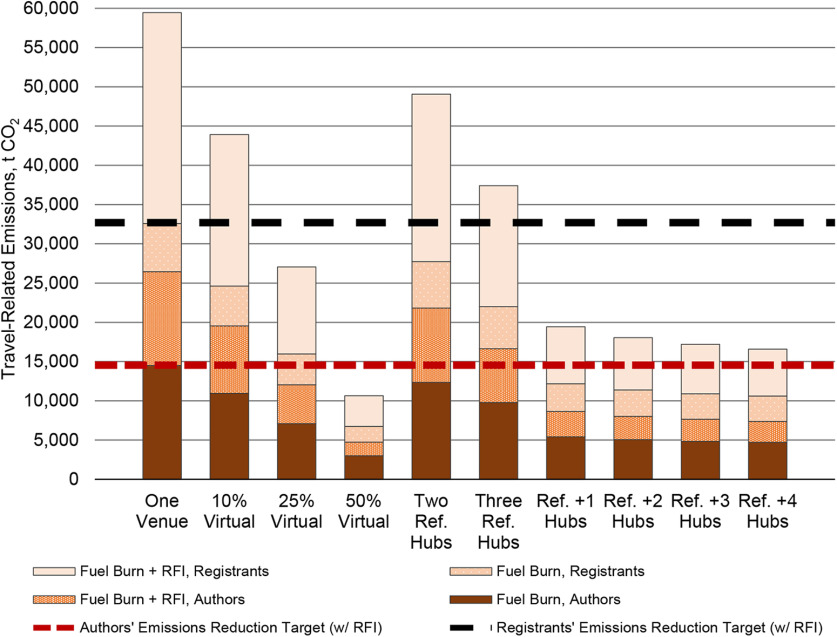
Summary of travel-related emissions for Neuroscience meetings by meeting mode. All estimations are derived from emissions rates in Jungbluth and Meili (2019). Radiative forcing index (RFI) factors account for an increased effect of global warming because of CO_2_ and non-CO_2_ emissions in the upper troposphere and lower stratosphere. Values for one venue, 10%, 25%, and 50% virtual are means across San Diego, Washington, DC, and Chicago meeting sites. Emission reduction targets are 55% of mean emissions for the three business-as-usual, in-person meeting sites. Three reference hubs include Frankfurt, Los Angeles, and Tokyo. Thereafter, we added, in order, Chicago, Dallas/Fort Worth, Mexico City, and Jakarta. Including emissions from connecting flights could increase emissions more than shown.

Four scenarios would significantly reduce Neuroscience annual meeting emissions by ≥45% before 2030, as per the IPCC (2018) recommendation (Fig. 7). First, SfN could incentivize ≥25% of the most distant registrants to attend virtually only, which may yield greater emissions reductions than randomly selected virtual attendance, as two studies have shown (Jäckle, 2021; Kuper, 2022). Registration rates for virtual attendance should be significantly less than in-person registrants, thereby making attendance more affordable and increasing participation by residents in more distant locations who would or could not attend otherwise. However, virtual attendees of hybrid conferences have fewer opportunities to engage fully with conference offerings. Second, SfN could convene in-person and virtually connect three reference hubs and one additional hub. While our use of Neuroscience 2018 authors in counterfactual scenarios does not account for attendance and travel mode differences by SfN members in proximal areas of hub locations, we found reductions of between 49% and 72% with six or seven locations. Third, until 2030 at the latest, SfN could meet biannually in person at a centralized location, as Nathans and Sterling (2016) proposed. Every other year, virtual or local meetings may convene. While this option may halve travel-related emissions, it does not address the behavioral change that will yield the greatest positive impact on the climate crisis. Ultimately, our estimations, and past research, indicate that only a completely virtual annual conference would reduce emissions close to 100% by 2050, relative to 2010 levels, as per the IPCC recommendation for 2050 (Tao et al., 2021; van Ewijk and Hoekman, 2021). Of these, the most equitable options are multihub and completely virtual conferences.

Concern among scientists regarding the impact of alternative meeting modes on science is understandable given that travel-related emissions among a large, random sample of researchers can be associated with a greater number of publications and higher h-index scores (Berné et al., 2022). However, multihub and virtual conferences may afford a greater number and a more diverse population of scientists access to collaborative opportunities than would otherwise be available with a centralized conference model. Deploying hub locations in category I, II, and III countries may yield even greater numbers of Neuroscience meeting registrants and members residing there. However, as DeRudder and Liu (2016) show, international attendance does not ensure international collaboration; intranational interactions and collaborations are more likely. Furthermore, multihub and virtual meetings could reduce travel distances and costs, particularly for Neuroscience meeting attendees residing in nations with lower-income economies. To better understand how SfN and Neuroscience meetings can be more inclusive, as Velin et al. (2021) recommends, SfN should conduct and make public an annual independent conference equity evaluation that may include investigating the degree to which Neuroscience meetings presentations are intranational and international.

While the authors recognize the value of conference attendance, and how motivations to do so may differ depending on career stage, humanity is at stake. SfN can and must respond immediately. Another business-as-usual Neuroscience conference adds to the existing emissions lingering aloft from past SfN conferences and contributes measurably to future global warming for at least 1000 years (Matthews and Solomon, 2013), the effects of which are irreversible for centuries to millennia (IPCC, 2023). Conversely, a rapid, radical reimagining of future SfN conferences that result in no greenhouse emissions contributes little to a warmer, uncertain future. To which future does the neuroscience community want to contribute?

## Synthesis

Reviewing Editor: Karl Herrup, University of Pittsburgh School of Medicine Decisions are customarily a result of the Reviewing Editor and the peer reviewers coming together and discussing their recommendations until a consensus is reached. When revisions are invited, a fact-based synthesis statement explaining their decision and outlining what is needed to prepare a revision will be listed below. The following reviewer(s) agreed to reveal their identity: anne-laure ligozat.

The authors have done an excellent job in responding to the concerns raised in the first round of reviews. Their thorough and thoughtful responses are welcome and reviewers and the Reviewing Editor are satisfied and have no further suggestions. A few very minor issue were noted that would benefit from attention. These are sufficiently small that a quick only a quick editorial review would be needed before passing the work on to publication.

- l. 61-63 : “A typical... persons” -> “A typical life sciences laboratory of 7 to 10 persons would “likely generate more than 20”t CO2 per year, or between 2 and 2.85 t CO2 per person”.

- l. 77: “Offering vegetarian ... percent” -> “Offering vegetarian or vegan menus could thus decrease emissions 1 to 2 percent only”

- l. 420 : “was associated” -> “can be associated” (may depend for example on the country of origin)

Finally, a comment from one reviewer that I share is to make every effort to make the raw data accessible. Even though it would require some work from the authors, it would be extremely useful to share the spreadsheets and software used in the study, so that other conferences can assess their carbon footprint too, and evaluate the impact of scenarios. These could be included as “Supplemental Material” with this manuscript.
