# Supplementary material for: Recommendations Emerging from Carbon Emissions Estimations of the Society for Neuroscience Annual Meeting
Source: eNeuro. 2023 Oct 12;10(10):ENEURO.0476-22.2023. doi: 10.1523/ENEURO.0476-22.2023 (PMC10580811; doi:10.1523/ENEURO.0476-22.2023)
Supplement: Table 2-1 — Neuroscience 2018 U.S. and Canadian presenting authors by region (n = 8278). Download Table 2-1, DOC file. [file enu-eN-NWR-0476-22-s06.doc]

Table 2-1. Neuroscience 2018 U.S. and Canadian presenting authors by region (*n* = 8,278).

| Region | State | Presenters |
| --- | --- | --- |
| Alaska | Alaska | 1 |
| Appalachia |  | 210 |
|  | Tennessee | 115 |
|  | Kentucky | 62 |
|  | West Virginia | 33 |
| Deep South |  | 618 |
|  | Georgia | 177 |
|  | Florida | 234 |
|  | South Carolina | 76 |
|  | Alabama | 67 |
|  | Louisiana | 43 |
|  | Mississippi | 21 |
| Desert Southwest |  | 185 |
|  | Arizona | 134 |
|  | New Mexico | 33 |
|  | Nevada | 18 |
| Great Plains |  | 552 |
|  | Texas | 473 |
|  | Oklahoma | 25 |
|  | Kansas | 22 |
|  | Nebraska | 17 |
|  | South Dakota | 10 |
|  | North Dakota | 5 |
| Mid-Atlantic |  | 2,159 |
|  | New York | 723 |
|  | Maryland | 526 |
|  | Pennsylvania | 390 |
|  | North Carolina | 183 |
|  | New Jersey | 170 |
|  | Virginia | 131 |
|  | Delaware | 36 |
| Midwest |  | 1,326 |
|  | Ontario, Canada | 281 |
|  | Illinois | 267 |
|  | Michigan | 189 |
|  | Ohio | 180 |
|  | Minnesota | 136 |
|  | Wisconsin | 116 |
|  | Indiana | 85 |
|  | Iowa | 72 |
| Mountain West |  | 125 |
|  | Colorado | 106 |
|  | Utah | 7 |
|  | Idaho | 6 |
|  | Wyoming | 4 |
|  | Montana | 2 |
| New England |  | 1,070 |
|  | Massachusetts | 541 |
|  | Quebec, Canada | 188 |
|  | Connecticut | 168 |
|  | Rhode Island | 78 |
|  | New Hampshire | 37 |
|  | Maine | 33 |
|  | Vermont | 14 |
|  | Newfoundland, Canada | 10 |
|  | New Brunswick, Canada | 1 |
| Ozarks |  | 126 |
|  | Missouri | 110 |
|  | Arkansas | 16 |
| Pacific Northwest |  | 415 |
|  | Washington | 160 |
|  | British Columbia, Canada | 93 |
|  | Alberta, Canada | 85 |
|  | Oregon | 77 |
| West Coast | California | 1,491 |
