# Supplementary figures and images for: Recommendations Emerging from Carbon Emissions Estimations of the Society for Neuroscience Annual Meeting
Source: eNeuro. 2023 Oct 12;10(10):ENEURO.0476-22.2023. doi: 10.1523/ENEURO.0476-22.2023 (PMC10580811; doi:10.1523/ENEURO.0476-22.2023)

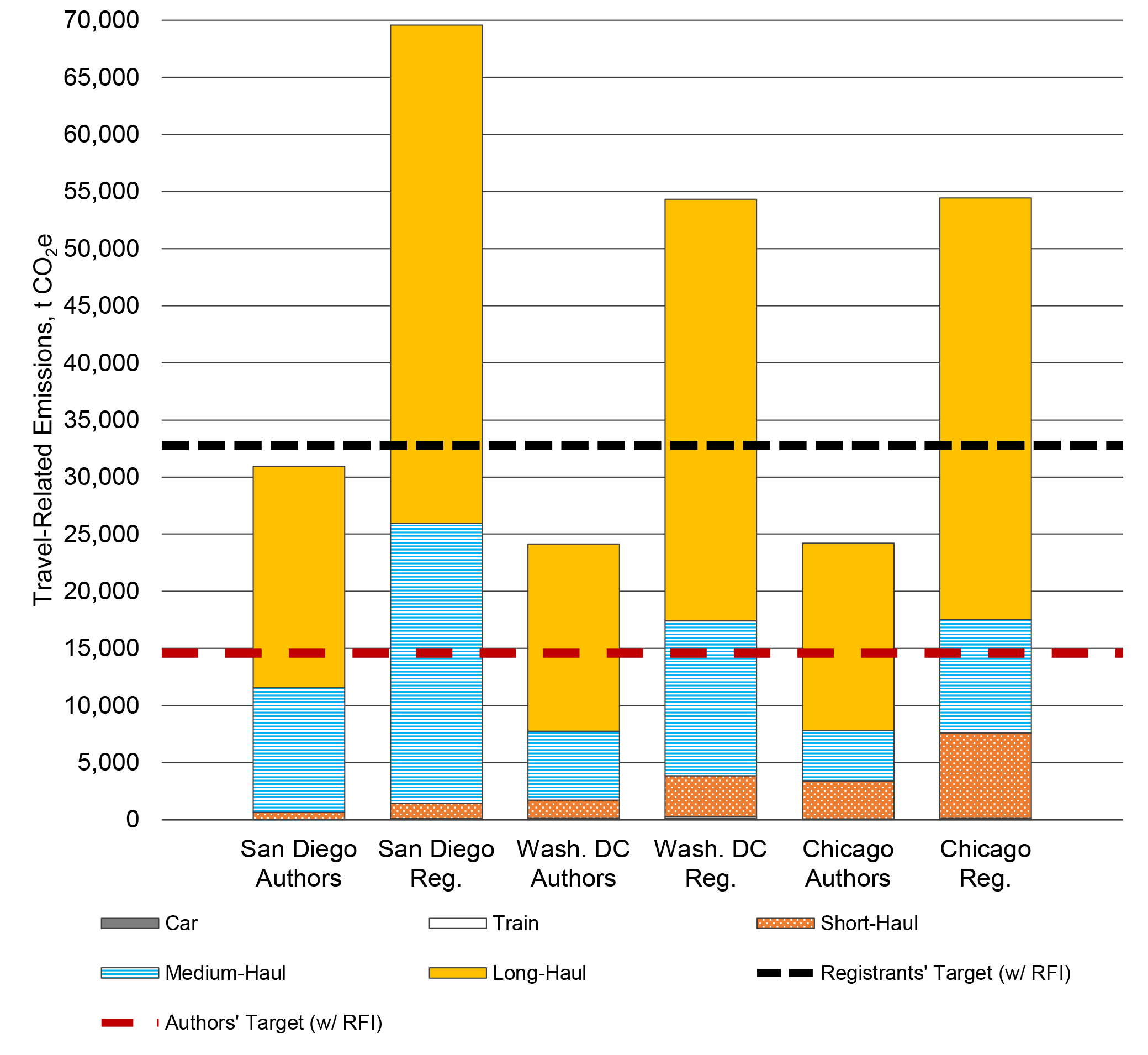

Supplement: Figure 3-1 — Travel-related emissions estimations, presented in t CO2e, derived from great-circle distances between Neuroscience 2018 presenting authors’ (N = 12,761) latitudes and longitudes and current, centralized meeting locations using emission rates from Jungbluth and Meili (2019). Also included are extrapolations for 28,691 registrants derived from mean emission rates and percentages of Neuroscience 2018 presenting authors by travel mode. Emission reduction targets of 45% are relative to mean travel-related emissions estimation across the San Diego, Washington, DC, and Chicago conference locations. Estimates for air travel include RFI factors that account for non-CO2 emissions, but do not include connecting flights, which could increase emissions more than shown. Authors’ emissions from cars for the San Diego, Washington, DC, and Chicago conference venues equaled 38.71, 45.17, and 20 t CO2, respectively; registrants’ emissions equaled 87.03, 101.56, and 44.96 t CO2, respectively. Authors’ emissions associated with train travel to Washington, DC and Chicago equaled 73.01 and 32.06 t CO2e; registrants’ emissions equaled 165.50 and 73.44 t CO2e. Download Figure 3-1, TIF file. [file enu-eN-NWR-0476-22-s02.tif]

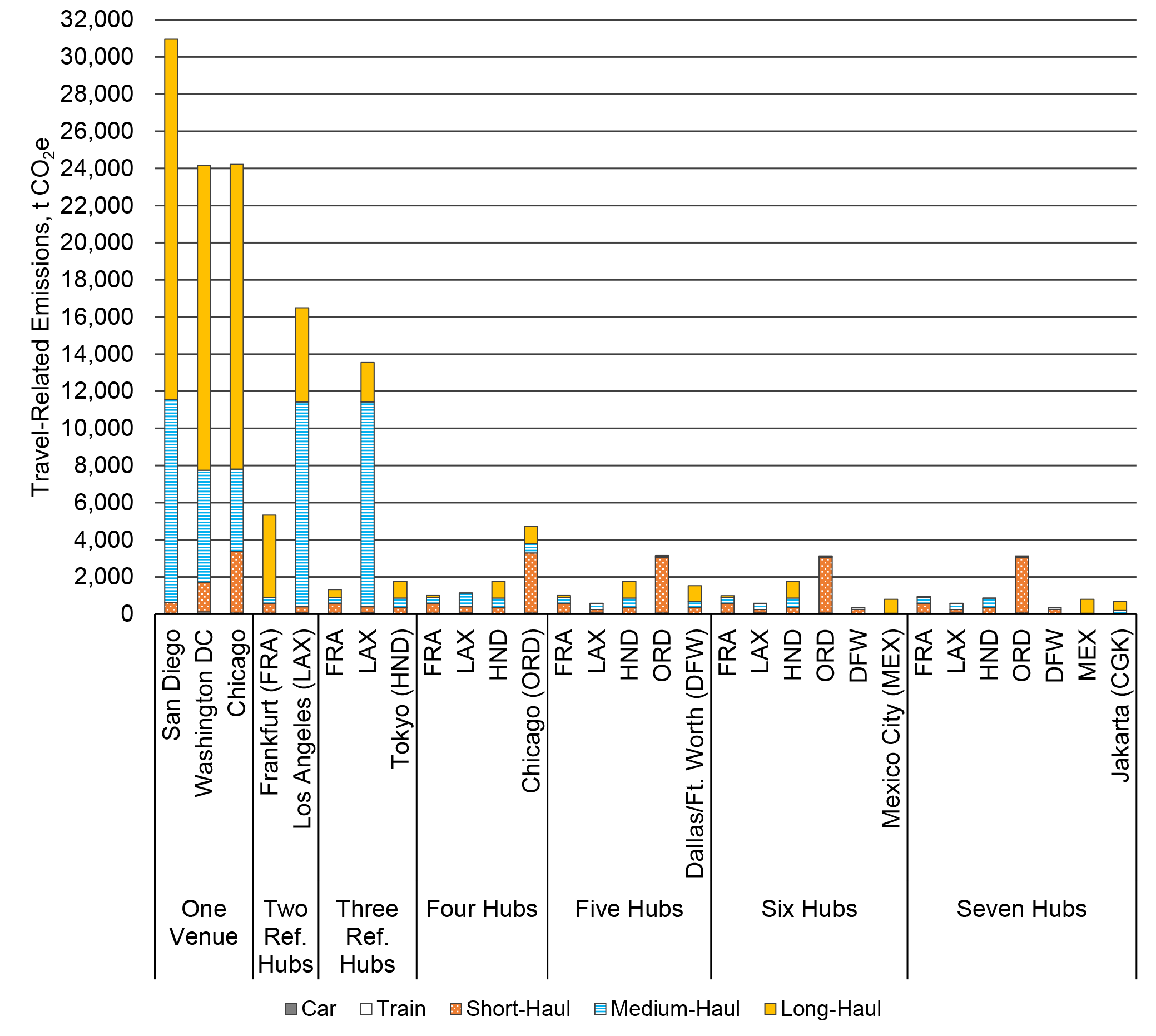

Supplement: Figure 4-1 — Counterfactual multihub meeting travel-related emissions, presented in metric tons of carbon dioxide equivalents (t CO2e), by travel mode, hub scenario and location derived from Neuroscience 2018 presenting authors (N = 12,761). Estimates include RFI factors that account for non-CO2 aircraft emissions. We did not account for connecting flights, which could increase emissions more than shown. Download Figure 4-1, TIF file. [file enu-eN-NWR-0476-22-s03.tif]

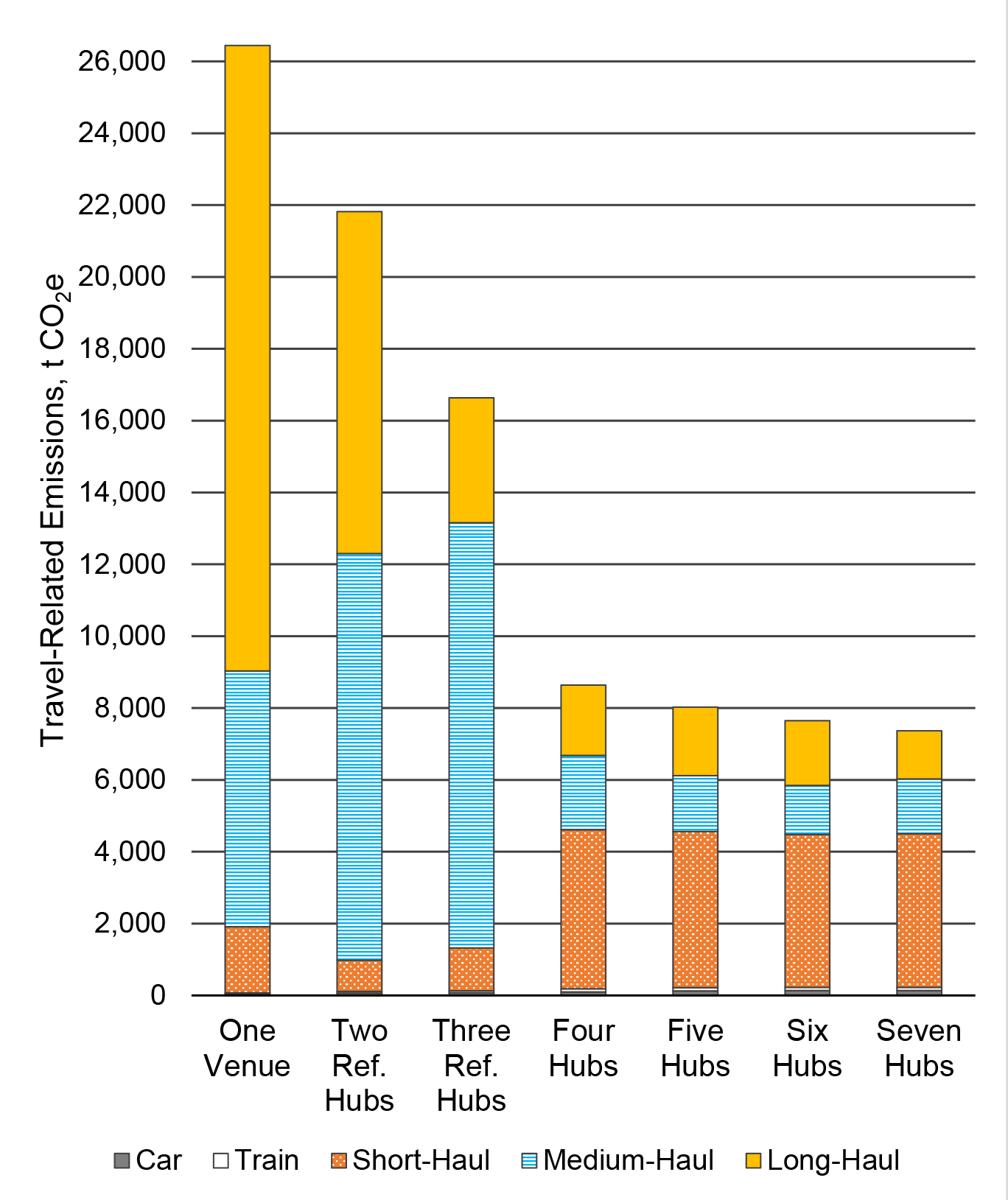

Supplement: Figure 4-2 — Summary of counterfactual multihub meeting travel-related emissions, presented in metric tons of carbon dioxide equivalents (t CO2e), by hub scenario and travel mode derived from Neuroscience 2018 presenting authors (N = 12,761). Estimates include RFI factors that account for non-CO2 aircraft emissions. Three reference hubs include Frankfurt, Los Angeles, and Tokyo. Thereafter, we added, in order, Chicago, Dallas/Fort Worth, Mexico City, and Jakarta. We did not account for connecting flights, which could increase emissions more than shown. Download Figure 4-2, TIF file. [file enu-eN-NWR-0476-22-s04.tif]

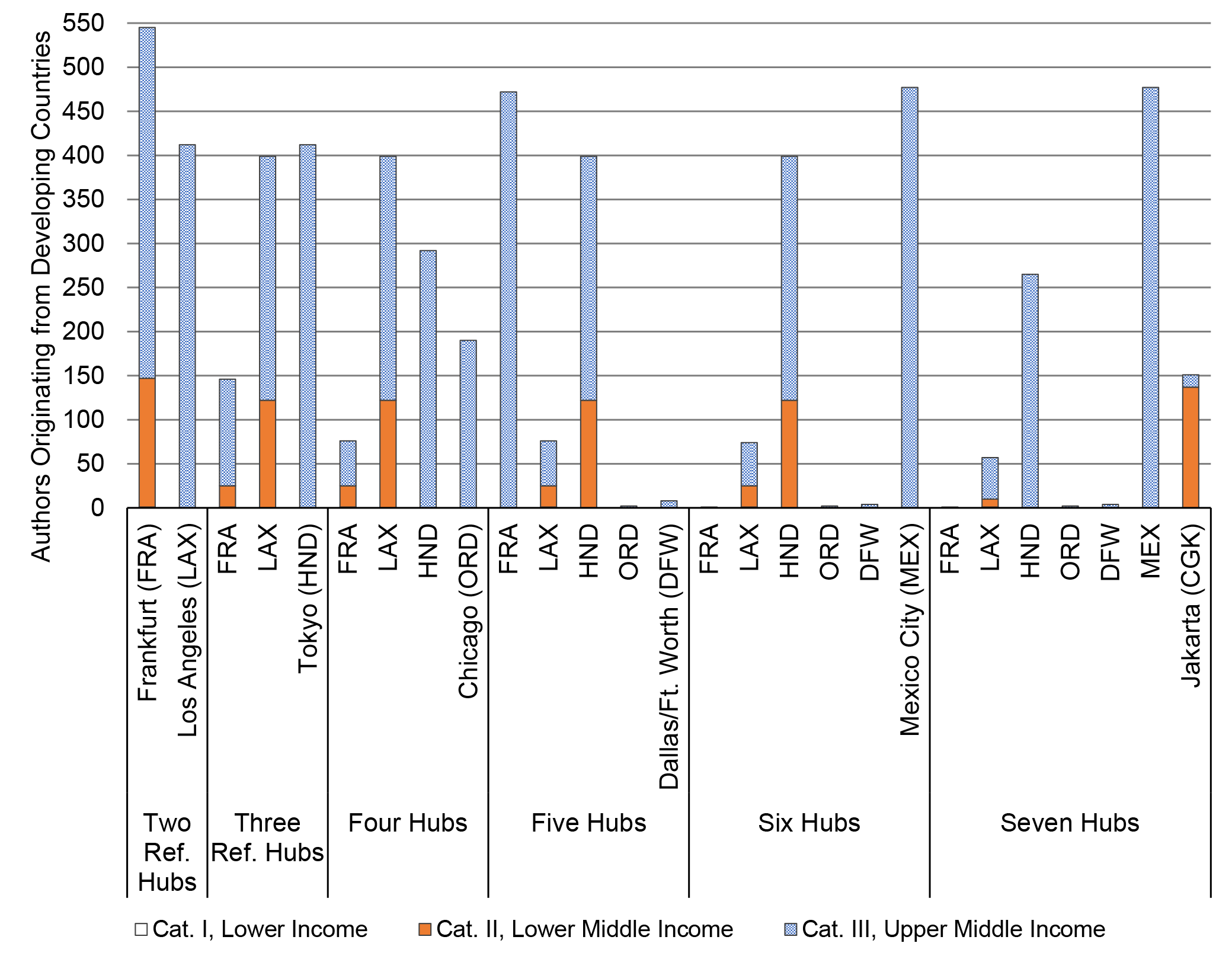

Supplement: Figure 5-1 — Attendance of Neuroscience 2018 presenting authors (n = 957) who originated from a developing country by World Bank income category in FY 2019, hub scenario, and location. Download Figure 5-1, TIF file. [file enu-eN-NWR-0476-22-s05.tif]
